# Patient-reported side effects and satisfaction of pre-hospital analgesia with low-dose esketamine: a cross-sectional study

**DOI:** 10.1186/s12873-023-00898-4

**Published:** 2023-11-04

**Authors:** David Häske, Fabian Eppler, Niklas Heinemann, Benjamin Schempf

**Affiliations:** 1 Center for Public Health and Health Services Research, University Hospital Tübingen, Osianderstraße 5, 72076 Tübingen, Germany; 2 Emergency Medical Service, German Red Cross, Steinenbergstraße 23, Reutlingen, 72764 Germany

**Keywords:** pain, emergency, paramedic, quality, safety

## Abstract

**Background:**

Analgesia is a core intervention in emergency medicine. Pain is subjective, so patient-reported experience with pain and analgesia is essential for healthcare professionals.

The aim of this study was to evaluate patient-reported side effects and satisfaction associated with pre-hospital analgesia with low-dose esketamine.

**Methods:**

This is an observational cross-sectional study conducted as part of quality assurance measures of the German Red Cross Emergency Medical Service, Reutlingen, Germany.

The survey was administered to all patients who received prehospital esketamine analgesia from paramedics. Addresses were obtained from medical records and mailed 10 days after the event. Patient feedback was anonymous and could not be linked to operational documentation.

**Results:**

A total of 201 patients were contacted, and 119 responses were received via the online questionnaire and postal mail (response rate 59%). The mean age of the patients was 68±13 years, with 64.7% (*n*=77) being female. The main diagnosis reported was fractures of the extremities in 69.7%. Patients reported initial median pain intensity on a Numeric Rating Scale (NRS) of 10 [8-10]. Pain was unbearable for 96.3% of patients. After administration of analgesia, 95.3% were satisfied or very satisfied. Patients reported no side effects in 78.5%, minor side effects in 10.0%, significant but well tolerable side effects in 11.3%, borderline tolerable side effects in 0.2%, and no unbearable side effects. Borderline tolerable nausea was reported in 2% of patients along with dreams in 0.8%. No nightmares were reported. Further analysis showed that patients older than 80 years reported significantly more side effects (*p* < 0.001) and were thus less satisfied with the analgesia.

**Conclusions:**

Both patient perception and analgesia with few side effects were important for both safety and satisfaction. In the present study, low-dose esketamine analgesia was associated with low side effects and high patient satisfaction.

**Supplementary Information:**

The online version contains supplementary material available at 10.1186/s12873-023-00898-4.

## Background

Analgesia is one of the most significant interventions in emergency medicine. Characteristics such as rapid onset, short-acting effect, good safety profile with few side effects are important from the perspective of patients and professionals [1, 2]. However, studies show that there are difficulties in pain assessment and that analgesia itself is inadequate and thus unsatisfactory for patients [3]. There are many reasons for this, both on the part of the practitioner and the patient [4]. For successful analgesia, it is therefore important to consider the patient's needs and involve them in the decision-making process, in addition to providing trustworthy patient care [5].

In addition, analgesia is dependent on the analgesic used. The most common analgesics in German (prehospital) emergency medicine are fentanyl, morphine, and (es-)ketamine, as well as sufentanil and metamizole [6, 7]. Piritramide and inhaled analgesics, on the other hand, are recently gaining ground in emergency medical service [8, 9].

Ketamine plays a special role here, as its effect is dose dependent. At low doses, esketamine is an analgesic; at higher doses, it causes dissociative anesthesia to an extent. The transition from analgesia to anesthesia depends on dosage, co-medication, and patient condition, among other factors. For ketamine analgesia, dysphoria, vivid hallucinations, and even "emergence phenomena" (or agitation) are consistently reported as side effects; fewer reports are available for esketamine, the S-enantiomer of ketamine [1]. Esketamine has its main effect primarily by blocking NMDA (N-methyl-D-aspartate) receptors and partially binds to the opiate receptor, with cardiovascular stimulation by catecholamine release, inhibiting peripheral reuptake of catecholamines, and central sympathetic stimulation.

The importance of the pharmacological quality of the drugs on the one hand, but especially the patients' perception of analgesic efficacy with low side effects is crucial for successful analgesia and high patient satisfaction. Particularly for low-dose esketamine, it is unclear how pronounced the side effects are and how much they interfere with patient satisfaction [6].

### Objectives

The aim is to assess the satisfaction and the side effects reported by the patients associated with analgesia with low-dose ketamine in a prehospital setting.

## Methods

### Study design

This is an observational cross-sectional survey study conducted as part of quality assurance measures of the German Red Cross Emergency Medical Service, Reutlingen, Germany. The reporting structure follows the Checklist for Reporting Of Survey Studies (CROSS) [10].

### Setting

Since 2005, the Emergency Medical Service (EMS) in Reutlingen qualified and authorized its paramedics in a competence system for the independent administration of esketamine analgesia according to the specifications of the medical director. Indications for esketamine include all moderate to severe pain (NRS ≥ 5, trauma-associated pain, low back pain, and abdominal pain) [11–13]. This advanced delegation is deemed appropriate training, standard operating procedure (SOP), along with annual competence checks [14]. Emergency medical services in Germany are organized by the state and are operationally dependent on the federal state and county. In addition to paramedics, who are trained for three years, it is also possible to send an emergency physician (doctor in training or specialist) to the scene of an emergency by air or ground in case of defined indications.

Low-dose esketamine analgesia in the studied EMS included the administration of esketamine (Esketamin Inresa^®^ 50mg/2ml) at an initial dose of 0.125 mg/kg body weight i.v. associated to 1 mg of intravenous midazolam (Midazolam Ratiopharm^®^, 5mg/5ml). Midazolam may be given once for dreams, psychomotor agitation, etc. Esketamine may be repeated as needed (inadequate pain relief, recurrent pain). Other analgesics have not been used.

### Questionnaire

Validated pain questionnaires exist mainly for chronic pain conditions (e.g., FQ-STAPM, DSF, etc.) [15, 16]. General patient satisfaction surveys, using validated questionnaires, are also available [17, 18], although surveys of pain perception and analgesia are less common [19, 20].

A good template has already been created by the working group of Sander et al. [20]. However, they focus more on satisfaction and less on side effects, so we developed our own questionnaire (Supplementary file). Question types used were binary response fields (e.g. bearable pain: yes/no), four-point scales (e.g. no pain, mild pain, moderate pain, severe pain) and eleven-point scales (e.g. Numeric Rating Scale 0-10), and an open field was used for general comments. We conducted nine pretests with four nonmedical citizens and five patients from the specified target population to optimize the questionnaire and test its comprehensibility.

### Data collection

The following data were collected: age, sex, previous experience with pain, pain assessment, patient-reported indication for analgesia, analgesia success, and incidence of side effects (Supplement questionnaire). The categories of side effects were developed based on various publications on esketamine analgesia and were classified as "no side effect, minor side effects, significant but well tolerable side effects, borderline tolerable side effects, unbearable side effects".

The pain-experience was measured with an endpoint named scale ranging from 0=no pain experience to 10=subjectively maximum pain experience.

Postal addresses were extracted from medical records and mailed 10 days after the event. In addition to the information text, the addressees received a paper questionnaire with a stamped return envelope and the option to complete the questionnaire online via a QR code. Patient feedback was anonymous and could not be linked to the operation or operation documentation or to specific patient data such as age, sex, body weight, or specific drug dosages.

### Sample characteristics

All patients who received esketamine with analgesia, administered by paramedics in the German Red Cross Emergency Medical Service of Reutlingen, Germany between June 02, 2022, and November 28, 2022, were included. There were no exclusion criteria given the inclusion criteria. For data collection, patients were contacted per mail 10 days after the event.

### Survey administrations

Questionnaires answered online via QR code were entered using Microsoft Forms. Multiple entries could not be intercepted. Postal returns were scanned using the optical character recognition software FormPro 3.1 (OCR-Systeme GmbH, Leipzig, Germany) and recorded as a dataset, with the correct composition and processing manually checked against the postal returns.

### Study preparation

This survey was preceded by various analyses of routine and quality assurance data, feedback from emergency physicians, paramedics and emergency department staff, and most importantly, feedback and interviews with patients.

### Statistical analysis

Descriptive statistics were reported with the metric scale for mean ± standard deviation (SD) when normally distributed or medians and interquartile range (IQR) when not normally distributed. Pearson's coefficient was used to describe correlations between metric variables. Frequencies are indicated with absolute and relative numbers. Two-tailed values of *p* < 0.05 were considered statistically significant. The χ2-test and, for independent samples with normally distributed data, the t-test or single-factor analysis of variance were used to calculate differences, or Mann-Whitney-U-Test when not normally distributed. Statistical analyses were performed with SPSS Statistics 28 (IBM, Armonk, NY, USA).

## Results

A total of 201 patients were contacted, with 119 responses received via online questionnaire and postal mail (response rate 59%). The mean age was 68 ± 13 years (range 12-87 years). 64.7% (*n*=77) of patients were female. The questionnaire was completed by 68.9% (*n*=82) of respondents, and 31.1% (*n*=37) completed by their relatives. When asked about their previous experience with physical pain with the question “How 'experienced' are you with severe physical pain, e.g., major injuries, illnesses, tumors, surgeries, or therapies? Ranked from "no pain experience" to "most severe pain experience" (e.g., kidney colic and severe injuries)” the mean response was 5.1 ± 2.5 on an 11-point Likert scale, which is a medium pain experience and interviewed patients obviously have quite painful experiences.

### Side effects of analgesia

**Fig. 1:**
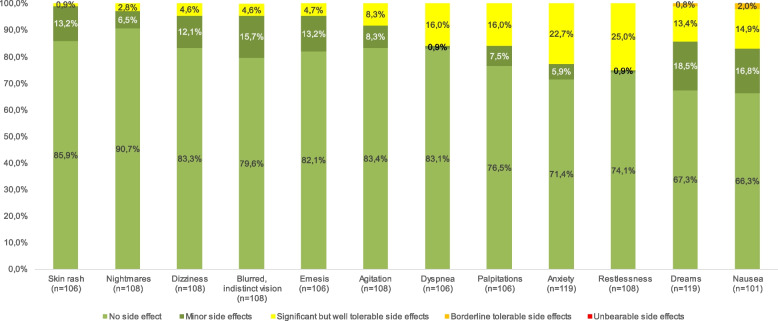
Frequencies of side effects reported. The graph shows the side effects on the x-axis and their frequency in % on the y-axis

Patients reported no side effects in 78.5%, minor side effects in 10.0%, significant but well tolerable side effects in 11.3%, borderline tolerable side effects in 0.2%, and unbearable side effects in 0% during low-dose analgesia with esketamine. Borderline tolerable side effects were nausea in 2%, followed by dreams in 1%. Significant but well tolerated side effects were restlessness in 25%, anxiety in 23%, palpitations and dyspnea in 16%. Dreams were also a significant but well-tolerated side effects in 13%. The three most frequent minor side effects were dreams in 19%, followed by nausea in 17% and blurred vision in 16%. The most common significant but tolerable side effect was restlessness with 25% (Fig. 1).

**Fig. 2:**
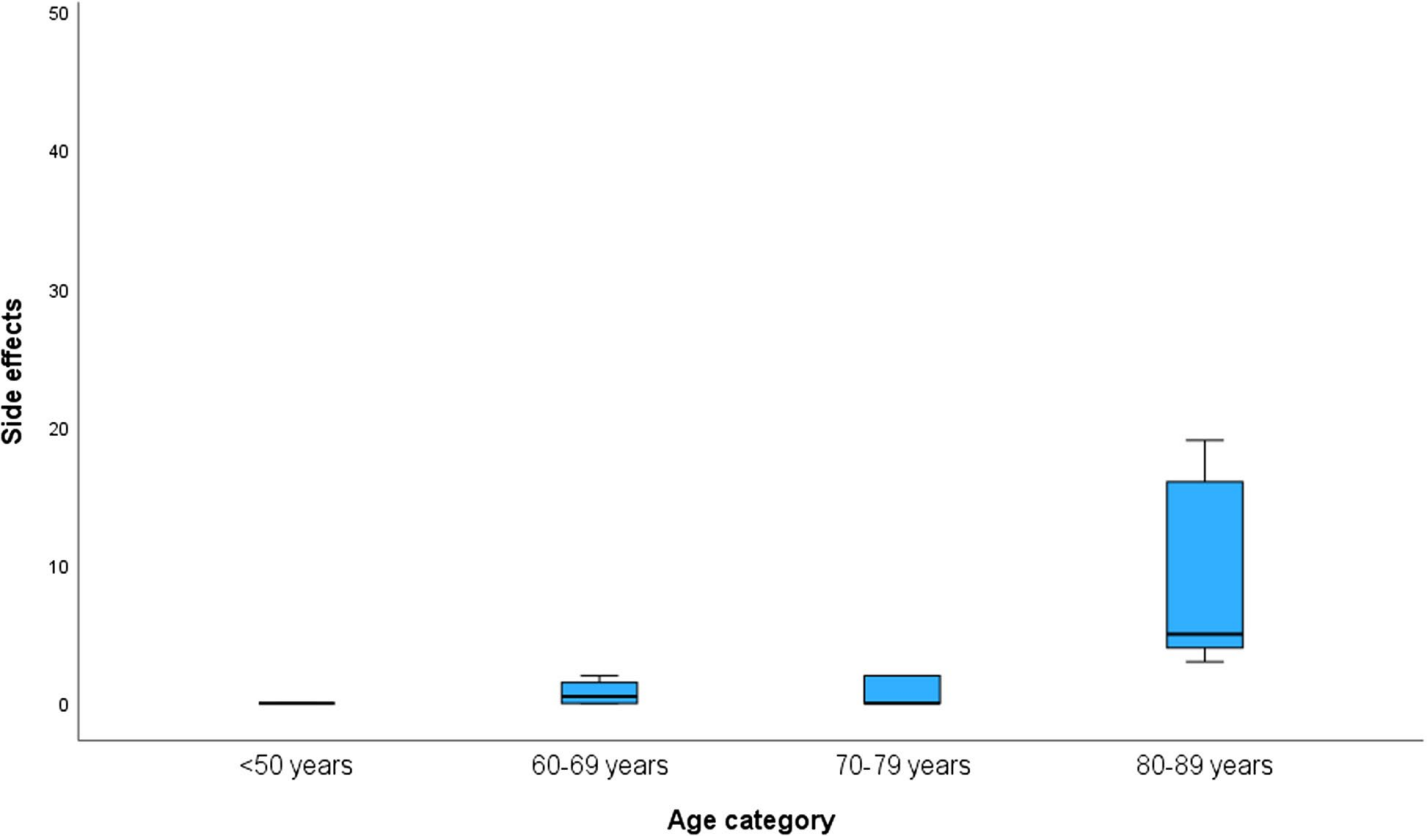
Patient-reported side effects in relation to age groups. If the severity (0=no side effects to 4= unbearable side effects) of the 12 side effects were summed, a total score between 0 and 48 would be possible. This shows that the age group <50 years reported no side effects, the age group 50-59 years was not represented, the age group 60-69 years had a median score of 0.5 [0-1.5], and the age group 70-70 years had a median score of 0 [0-2]. In contrast, the oldest age group, 80-89 years, had a median score of 5 [4-16]

Analysis showed that patients ≥ 80 years reported significantly more side effects (*p*<0.001) (Fig. 2).

### Indication and pain assessment

Fractures were the cause of pain and the indication for administration of analgesia in 69.5% of patients. Not all reported indications for analgesia were obvious, such as dyspnea (*n*=6, 5.1%) (Table 1).

**Table 1:** Reported indication for analgesia

**Indication**	**N (%)**
**Fractures**	**82 (69.5%)**
Extremities	74
Thorax	8
**Dislocations**	**14 (11.9%)**
Extremities	14
**Urinary retention**	**8 (6.8%)**
**Contusion**	**7 (5.9%)**
Extremities	7
Dyspnea	**6 (5.1%)**
**Unclear**	**1 (0.8%)**
Abdomen	1
**Overall result**	**118 (100%)**

The Question was “Why did you receive a painkiller? What was your diagnosis/condition/injury?“ Grouped patient feedback

Patients reported initial median pain on an NRS of 10 [8-10] (range 5-10). The additional question of whether the pain was considered tolerable prior to treatment was answered in the negative by 96.3%. When asked hypothetically if they could sleep with the pain, 96.6% (*n*=115) answered no. On a four-point pain scale (no pain, slight pain, moderate pain, severe pain), 6.5% (*n*=7) of patients reported moderate pain and 93.5% (*n*=101) of patients reported severe pain. Patients in the "moderate pain" group reported a median pain NRS of 6 [5-7], while patients in the "severe pain" group reported a median pain NRS of 10 [8-10]. A total of 104 patients (92.9%) received additional analgesia in the hospital. Following the administration of analgesia in the prehospital setting, 95.3% of patients were satisfied or very satisfied.

**Fig. 3:**
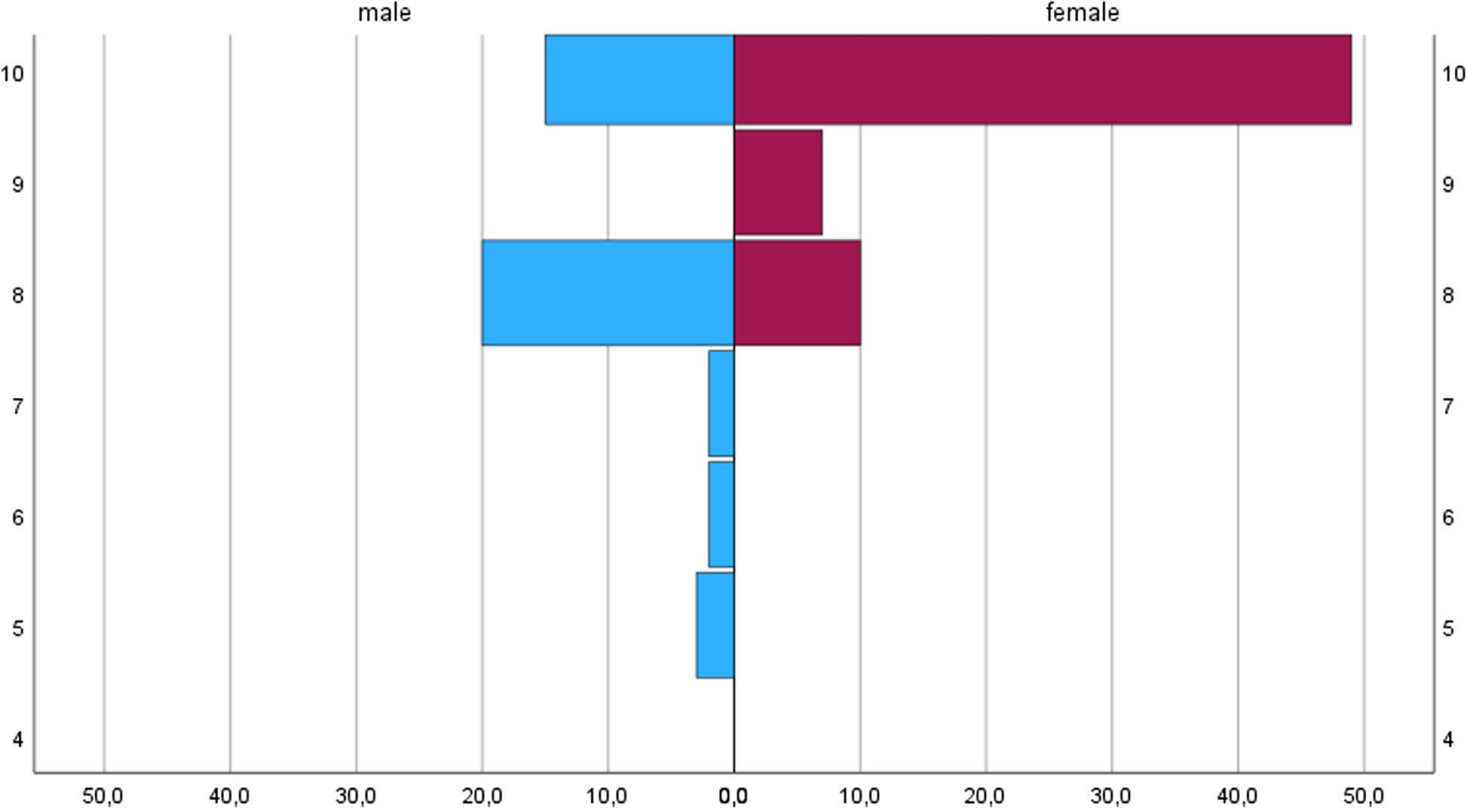
Pain in function of the gender. This figure shows the pain score on the numeric rating scale (NRS) on the vertical axis and the frequencies of pain in % for men and women on the horizontal axis

Pain experience was significantly correlated with increasing patient age (*r*=0.674, *p*<0.001). A correlation between "pain experience" and initial pain on the NRS could not be shown (*r*=0.030, *p*=0.758). When comparing the pain rating in function of the gender, female patients reported a higher NRS compared to the male (median NRS 10 [9-10] versus 8 [8-10], *p*<0.001) (Fig. 3).

Satisfaction with esketamine analgesia decreased significantly (*p*<0.001) in this age group (Table 2).

**Table 2:** Patient satisfaction

		Was the pain therapy appropriate to the situation and, in your opinion, sufficient?
		Median [Interquartile range]
Age category	<50 years	4.5 [4.0-5.0]
	50-59 years	5.0 [5.0-5.0]
	60-69 years	5.0 [5.0-5.0]
	70-79 years	4.3 [4.0-5.0]
	80-89 years	4.0 [4.0-4.0]
	≥90 years	.

Patient satisfaction in this question is composed of the two characteristics “appropriate” and “sufficient” and is presented here as a function of age using mean and standard deviation on a 5-point Likert scale

## Discussion

Good patient perception and low side effects of analgesia administration in a prehospital setting are very important for patient safety and satisfaction for patients and provider with analgesia. In the present study, analgesia with low-dose esketamine was associated with low side effects and high patient satisfaction in several indications.

### Side effects

Patients interviewed reported analgesia with generally few side effects, never reported unbearable side effects, but borderline manageable nausea in 2%. The literature reports nausea with ketamine in approximately 1% of patients [6]. However, dosage is not well known, nor is patient age. It is also not possible to deduce from patient feedback if the nausea was preexisting or a result of analgesia. Influence of distance and duration of transport needs to be discussed, including "backward" position. Similarly, anxiety may have been due to the emergency, rather than the analgesia.

The second borderline tolerable side effect was "dreams" but not nightmares at 0.8%. The distinction was made between positive and negative "dreams". The classification used shows a weakness because the interpretation of "borderline tolerable dreams" is somewhat difficult. Ketamine in particular has been reported to cause frequent psychic reactions [21]. However, it seems difficult to predict which patients are more likely to experience neuropsychiatric side effects, which can range from dysphoria and vivid hallucinations to agitation [6, 22]. The reported rates vary from 5 to 30% [21]. In our study, with the lower side effect of low-dose esketemine compared to ketamine, negative dreams occurred in less than 3% and were also described as "tolerable". In contrast, motor agitation, which was well tolerated, was reported in 25% of patients. Benzodiazepines are usually recommended in advance to reduce psychomotor reactions [21]. However, studies indicate that psychogenic effects occur only during the ketamine offset phase [23–26]. Interestingly, also the package information leaflet for esketamine (Inresa) states that "the risk of psychiatric reactions on awakening from anesthesia may be greatly reduced by combination with a benzodiazepine", suggesting that this phenomenon may be related to the offset phase and may not be equally pronounced and thus relevant to therapy [27]. Thus, it is debatable whether benzodiazepine should be routinely added to low-dose esketamine or selectively added as needed.

We found also that older patients reported significantly more side effects than younger patients. Additionally, patients in the same subgroup (≥80 years) were significantly more dissatisfied than others, but this is an extremely relevant point for emergency medicine. There are limited data on ketamine in the context of analgesia in geriatric patients. A study comparing an intravenous subdissociative dose of ketamine with morphine for analgesia in geriatric patients showed comparable analgesia with higher rates of psychoperceptual side effects [28]. A slower metabolism in elderly patients was discussed [29]. A study of low-dose esketamine for induction of anesthesia in elderly patients undergoing knee arthroplasty again showed no adverse events in the recovery period [30].

### Indication

The indication for esketamine, or ketamine, is often associated with prehospital trauma pain or analgesia and anesthesia of polytrauma [31, 32]. Ketamine is fast-acting, easily controlled, and has primarily beneficial effects on the cardiovascular system with the consequent property of supporting some circulatory stability, which is countered by increased myocardial oxygen consumption. However, the clinical use of (es)ketamine is much broader: (es)ketamine can be found in the entire perioperative field, including regional anesthesia for caesarean section, but also, for example, breakthrough pain in herpes zoster, palliative situations [33–35]. In pediatric emergency analgesia, it offers a wide range of application options, with intranasal use [27]. Esketamine currently plays a minor role in emergency departments, although low-dose ketamine, also in the form of ketamine infusion, is becoming increasingly popular [36]. Ultimately, (es)ketamine can be used for pain that is difficult to relieve with conventional medications, taking into account contraindications [37].

However, the indications for analgesia in the present study are interpreted by the patients. Injuries, without reference to the diagnoses that are usually not known with certainty in the prehospital setting, are also relatively well reflected as some indications by patients. Some indications are less plausible. For example, the indication of dyspnea was reported by 6% (*n*=5), but it is not possible to determine what might have caused the dyspnea, such as thoracic trauma, because there is no link to the case documentation.

### Patient satisfaction

When gender was compared with the mean pain reported on arrival at the EMS, significantly more women reported severe or very severe pain. This result is unadjusted and comes from a small number of cases, but the literature also shows that women have more severe pain than men [38]. Reasons for this include biological and psychosocial processes but also stereotypical gender roles [39]. However, this may become more important in the future, both in terms of pain assessment and analgesia concepts.

It was expected that pain experience would correlate with increasing age or life experience, and this is significantly reflected. The hypothesis that patients with low pain experience might rate their pain higher than patients with high pain experience could not be confirmed here. This might have required a different study design and a larger sample size.

### Limitations

The main limitation of this study is due to the chosen methodology and lack of linkage between patient feedback and operative documentation. The authors are aware of this, as well as limitations due to the sample size and the study’s descriptive nature. As such, the results are not necessarily generalizable, but represent an insight into the findings of the collective studied.

Although the patients' feedback sounds largely plausible, it must be taken into account that the patients were given a sedative for each analgesia and may not remember it correctly. Approximately 30% of respondents were assisted in completing the questionnaire, which can introduce bias. Although a dose of 1 mg of midazolam is not considered high, it may still impact the experience.

The time between the event and questionnaire delivery was 10 days. Some feedback criticized that the period was too long, while other feedback found it to be too short. Theoretically, a distortion due to positive event recall bias could be discussed, influencing the perception of care and side effects.

What the survey does not show are critical events such as respiratory depression and similar medical issues. No adverse events were seen in operational documentation during the data extraction review.

## Conclusion

Thus, the use of low-dose esketamine for pain management in the prehospital setting appears to be associated with very high patient satisfaction, with few reported side effects. Also, esketamine appears to be satisfactory in all age groups in the studied collective; however, from this, patients over 80 years of age should be treated more cautiously to minimize side effects while achieving adequate analgesia. In this age group, other treatment options (e.g., opioids, but also inhaled analgesics) may have better treatment pathways in the elderly.

### Supplementary Information

**Additional file 1.**

## Data Availability

The data sets analyzed in the current study are available for on-site review upon request from the corresponding author.

## Abbreviations

EMS: Emergency Medical Service
NRS: Nummeric Rating Scale
PROM: Patient-reported Outcome Measure
