# Supplementary material for: Patient-reported side effects and satisfaction of pre-hospital analgesia with low-dose esketamine: a cross-sectional study
Source: BMC Emerg Med. 2023 Nov 4;23:130. doi: 10.1186/s12873-023-00898-4 (PMC10625244; doi:10.1186/s12873-023-00898-4)
Supplement: Supplementary file 1 — Additional file 1. [file 12873_2023_898_MOESM1_ESM.pdf]

DRK Kreisverband Reutlingen
Rettungsdienst

Deutsches Rotes Kreuz

Patient survey 2022 - How satisfied were you with us?

Dear patients,

We would be pleased if you could take a few minutes to answer the following questions.

1. **Why did you receive a painkiller? What was your diagnosis/condition/injury?**

____

2. **How did you experience the wait time until the arrival of the emergency medical services?**

very long	long	average	short	very short	No memory
☐	☐	☐	☐	☐	☐

3. **Could you have waited longer for pain medication given your pain?**

Yes	No	No memory
☐	☐	☐

4. **Do you know who administered the pain medication to you (Doctor, paramedic, EMT, etc.)?**

____

5. **How satisfied were you with the information/explanation regarding the pain treatment?**

☺☺	☺	😐	☹	☹☹	No memory
☐	☐	☐	☐	☐	☐

6. **Was the pain management appropriate for the situation and, in your opinion, sufficient?**

☺☺	☺	😐	☹	☹☹	No memory
☐	☐	☐	☐	☐	☐

translated questionnaire

7. **Did you receive additional pain medication at the hospital/emergency room?**

Yes	No	No memory
☐	☐	☐

How did you perceive your pain?

8. **How would you rate your pain upon the arrival of the emergency medical services on a scale from 0=no pain to 10=maximum pain?**

0	1	2	3	4	5	6	7	8	9	10	No memory
☐	☐	☐	☐	☐	☐	☐	☐	☐	☐	☐	☐

9. **Could you tolerate your pain before the treatment (well)?**

Yes	No	No memory
☐	☐	☐

10. **Hypothetically, could you have slept with this level of pain?**

Yes	No	No memory
☐	☐	☐

11. **How would you rate your pain upon the arrival of the emergency medical services?**

No pain	minor pain	moderate pain	severe pain	No memory
☐	☐	☐	☐	☐

translated questionnaire

Deutsches Rotes Kreuz

Side Effects

12. Did you experience any side effects?

	No side effects	mild side effects	significant but good manageable side effects	barely tolerable side effects	unbearable side effects	No memory
Nausea	☐	☐	☐	☐	☐	☐
Vomiting	☐	☐	☐	☐	☐	☐
Shortness of breath	☐	☐	☐	☐	☐	☐
Rash	☐	☐	☐	☐	☐	☐
Palpitations	☐	☐	☐	☐	☐	☐
Anxiety	☐	☐	☐	☐	☐	☐
Dizziness	☐	☐	☐	☐	☐	☐
Vivid dreams (positive)	☐	☐	☐	☐	☐	☐
Nightmares (negative)	☐	☐	☐	☐	☐	☐
Restlessness	☐	☐	☐	☐	☐	☐
Muscular restlessness	☐	☐	☐	☐	☐	☐
Blurred or unclear vision	☐	☐	☐	☐	☐	☐

How were your experiences with the German Red Cross ambulance service?

13. Was our team polite and friendly?

☺☺	☺	😐	☹	☹☹	No memory
☐	☐	☐	☐	☐	☐

14. Was our team empathetic regarding your situation of distress?
15.

☺☺	☺	😐	☹	☹☹	No memory
☐	☐	☐	☐	☐	☐

16. Did the team convey a competent impression?

☺☺	☺	😐	☹	☹☹	No memory
☐	☐	☐	☐	☐	☐

translated questionnaire

17. Did you feel safe, well taken care of, and supported?

☺☺	☺	😐	☹	☹☹	No memory
☐	☐	☐	☐	☐	☐

At the end, a few questions about you:

18. How old are you?

________________________________**Years**

19. To which gender do you identify?

female	male	diverse	prefer not to say
☐	☐	☐	☐

20. How experienced are you with severe physical pain, such as significant injuries, illnesses, tumors, surgeries, or treatments?

Barely experienced pain				⇔					*Experienced the most severe pain (e.g., kidney stones, serious injuries*
☐	☐	☐	☐	☐	☐	☐	☐	☐	☐

21. Who is filling out the questionnaire?

☐ Patient ☐ Family Member ☐ other:________________________

translated questionnaire

Deutsches Rotes Kreuz

22. other comments:

Thank you very much for participating in our survey. We are very committed to using the best materials and state-of-the-art technology for the benefit of our patients. However, our highly competent rescue teams are particularly important to us, as they undergo annual training, further education, and testing to provide you with the highest level of care.
We wish you all the best and a speedy recovery.

Yours sincerely
DRK Kreisverband Reutlingen
Formularbeginn
Formularende

Kontakt:
Email: qm@rettungsdienst-reutlingen.de
Telefon: 07121 9287-0

translated questionnaire
